# A Case Showing the Danger of Error in Diagnosis

**Published:** 1873-10

**Authors:** Milton G. Potter

**Affiliations:** Professor of Anatomy in Buffalo Medical College


ART. II.—*A Case showing the Danger of Error in Diagnosis.* By MILTON G. POTTER, M. D., Professor of Anatomy in Buffalo Medical College.

On the night of October, 3, 1870, I was called to attend Mrs. O. in confinement, she being at the time of my visit a perfect stranger to me. She was delivered at 4 A. M., Oct. 4th, of a male child, after a natural labor of about four hours duration. This was her first confinement. During the labor she complained bitterly of pain in the left side, and also of pain in the left thigh. This pain, however, in the thigh and side, had been a source of constant complaint during the period of her pregnancy, and the pain in the side had been frequently present during the last two years. The afterbirth was detained about half an hour, when it was delivered without special aid from me. The labor was a remarkably clean one; the expulsion of the placenta being accompanied with no clots and no hemorrhage. Upon applying the bandage I was impressed with the large size of the abdomen and the unusually high position which the womb retained. It was, however, itself not of abnormal size. Assuring myself that my patient was not still pregnant, I made inquiry about her natural size, and was informed that during the year preceding her pregnancy, she had been unpleasantly large. Her bowels had moved freely twice during the night, the result of a cathartic she had herself taken, and she had also made water, both before and during my visit. Her pulse was 74, and as her womb was well contracted, I felt safe in leaving her, though I confess I did not have any well defined reason for her size. At 4 P. M., twelve hours after the delivery of Mrs. O., I was summoned, in great haste, to see her servant, who had been flowing profusely. She had already fainted, and was in syncope when I arrived. Examination showed the womb above the pubis, the vagina occupied by large clots, and the os open and flaccid. The servant was at once given Brandy, Ergot and Morphine and a bandage tightly applied. She confessed that she had had a miscarriage; had herself taken the fœtus out of doors; returned to her room; then delivered the placenta, carried that also out of doors; had again returned to her room, and that she had flowed continuously from the time she started with the afterbirth until she fainted after her return to her bed. The condition of the rooms through which she had passed, (one of which was the room occupied by Mrs. O.), was abundant proof of her confession. At this time Mrs. O. is found considerably excited and tremulous—has not slept—is very much annoyed, fearing that she will again see the girl. She will not be comforted, but is startled by every noise, and thinks that that "terrible girl" is again coming through the room. Her pulse is 84 and she has urinated freely, using the bed-pan. ℞ Morphia Sulph. gr. ss., repeat in two hours if not quiet. 8 P. M.—Mrs. O. still excited; has taken a grain of Morphine since 4 P. M., and again urinated in the bed-pan. The discharges from her are thought about normal in quality and amount. She is so large, however, that I am induced to reapply the bandage and examine the abdomen more closely than I have hitherto done. The womb, which could be felt distinctly before and after the expulsion of the placenta, cannot now be so clearly defined. Its fundus appears above the umbilicus, but its body is concealed behind a hitherto unrecognized tumor. The tumor is found to fluctuate and occupies the portion of the abdomen, between the umbilicus, symphysis, pubis and crest of left illium. Without making the existence of this tumor known, I enquired if the patient had ever had any trouble in the region occupied by it. She replied that she had suffered a great deal of pain there during the last two years; that in the Summer of 1868, when it began, she was very sick for about two weeks, having, as her physician said, inflammation in this region, and, further, that she could, since this sickness, frequently feel a tumor there as large as an orange, and as frequently it would disappear and could not be felt. It would come and go. She had felt it, and had a great pain in it during her pregnancy. It had not before been examined by a medical man. It was at this time as large as a fœtal head. Diagnosis unilocular cryst of left ovary Pulse 80.

℞ Pulv Opii, gr. vi.
Eng. Ext. Hyoscyami.
Pulv. Camphoræ, aa gr. xviii.
Ft. pilulæ No. XII.
Take one every two or four hours, as needed.

October 5, 8 A. M.—Patient has not slept any during the night, and is much excited because her servant is still in the house. She has passed water involuntarily to some extent during the night, and, after a voluntary effort, urinated on cloths early in the morning. Her left leg is considerably swollen, and pits upon pressure. The abdomen is so large that I removed the bandage and re-examined it. The right lateral half of the abdomen is considerably larger than natural and tympamtic. The left lateral half is larger the right, but dull and even flat upon percussion. Fluctuation is again recognized. Fearing still that the womb may be in some manner occupied, I request a vaginal examination. While I am preparing to make this the patient voids a considerable quantity of urine. The finger applied against the os reveals nothing abnormal, except that it is very high up. The pelvis is apparently free. The fundus can be felt with the left hand on the abdomen, while the right forefinger is against the os. The fluctuating cyst was thus found to be neither a part of the womb nor connected with it, and the diagnosis already expressed was confirmed to my entire satisfaction. Her pulse was 82 and she was flowing about normally.

℞ Potassii Bromidi ℈ii every two hours, with Pulv. Opii gr. iss, if not quieted by the third dose of the Bromide.

Oct. 5, 9 P. M.—The servant girl has been removed to the hospital. My patient has been sleeping. Pulse 82. Continue treatment.

Oct. 6, 9 A. M.—Slept well during the night and is greatly refreshed thereby. The milk is coming and the child is regularly applied to the breast. After pains are present. She has control over urination and has made water freely. Whole aspect much improved. Pulse 72 and soft. Pain is only complained of in the tumor and left leg. The abdomen is still terribly distended and there have been during the night frequent eructations of gasses from the stomach. Her hearing is now considerably impaired, though she has taken no Quinine.

℞ Bismuthi Sub. Carb., gr. v., every three hours, with anodynes, as required.

Oct. 7, 9 A. M.—Has been very comfortable since last report, Her sleep during the night was somewhat interrupted, probably from the pain and annoyance which the escape of gases from the rectum and stomach occasions; complains of distension in the rectum and of general abdominal fullness. Has urinated normally. Pulse 76.

℞ Olei Ricini, ℥ss.

Oct. 8, 9 A. M.—Bowels moved twice yesterday, with relief. She has passed a comfortable night. Urine, however, has again come involuntarily from her several times in small amounts. She has made water once by voluntarily effort during the night. Pulse not recorded. Is seen by Dr. Smith.

1 P. M.—Am summoned on account of a chill. She has pain in her leg and over her womb. Pulse 120; abdomen larger; urinates involuntarily; has just taken a domestic dose of whiskey and is much excited.

℞ Morphine Sulph., gr. ss. at once; repeat in gr. ¼ doses every two hours till tranquil. Also Quinine, gr. iii, every 4 hours, with beef tea and brandy; hop fomentations over the abdomen.

6 P. M.—Has become quiet; pulse 112.

9 P. M.—Condition unchanged; continue treatment; the lochia have ceased.

Oct. 9th, 9 A. M.—Pulse 114. Urinates involuntarily, but in amount sufficiently large.

10 P. M.—Lochia re-established, though pulse are 120.

Oct. 10.—Continues much the same. Fomentations are omitted and the bandage reapplied. Throughout the remainder of her sickness she experienced great relief from the bandage when tightly applied. The history of this patient from October 10th to October 18th, the day of her death, is one of fatal puerperal metritis, with nothing in it of special interest save that, with three exceptions, she urinated involuntarily during these seven days. The amount, however, passed during each day was regarded, both by the nurse and myself as abundant. She was seen three times by Dr. Smith, once by Dr. Rochester, and once by Dr. Potter, of Lancaster. Dr. Rochester was particular in his examination with reference to urination, and was abundantly satisfied that she made water enough. Dr. Smith first saw the patient, Oct. 8th, when the tumor could be well defined, and concurred with me in my diagnosis.

Dr. Rochester saw the patient, Oct. 15th, when her condition was such as to render an examination for ovarian tumor improper, though the well marked dullness in the side, and my history of it, led him to suspect its existence.

The patient died at 4 A. M., Oct. 18th.

*Post-mortem*, 8:30 P. M., same day, in presence of Drs. Rochester, Smith, Strong, and myself. Rigor mortis is not well pronounced; abdomen is still terribly distended; percussion shows tympanitis and dullness as before death. Upon opening the abdomen no fluid is found in the peritoneal sac, nor was there any evidence of inflammation of that membrane. The intestines are so full of gases that they roll out and almost conceal the tumor. By puncture these are made to collapse, and the cyst is exposed. It occupies the left lateral half of the abdomen, nearly as high as the umbilicus, but does not extend much beyond the linea alba. It is found not to look exactly like an ovarian cyst; it is not full of fluid; its walls are not tense; its contents are very fluid. On this account we are led to examine the sac more closely. It is found attached to neither ovary nor womb; but, by a broad surface, the side of the sac is firmly adherent to the plane of the Ischium and the attachment tapers as it reaches the pectineal line. Below, it is continuous with the urethra, and we find no other bladder except it. It contains between four and five quarts of urine and the walls do not collapse after evacuation. The walls are thin. The uterus presented the usual marks of inflammation.

